# Favipiravir Inhibits Mayaro Virus Infection in Mice

**DOI:** 10.3390/v13112213

**Published:** 2021-11-03

**Authors:** Michèle Bengue, Ai-rada Pintong, Florian Liegeois, Antoine Nougairède, Rodolphe Hamel, Julien Pompon, Xavier de Lamballerie, Pierre Roques, Valérie Choumet, Dorothée Missé

**Affiliations:** 1 MIVEGEC, Univ. Montpellier, IRD, CNRS, 34394 Montpellier, France; francemich7@gmail.com (M.B.); ai-rada.pintong@ird.fr (A.-r.P.); florian.liegeois@ird.fr (F.L.); rodolphe.hamel@ird.fr (R.H.); julien.pompon@ird.fr (J.P.); 2 Unité des Virus Emergents (UVE), Institut de Recherche pour le Développement 190, IHU Méditerranée Infection, Institut National de la Santé et de la Recherche Médicale 1207, Aix Marseille Université, 13005 Marseille, France; antoine.nougairede@univ-amu.fr (A.N.); xavier.de-lamballerie@univ-amu.fr (X.d.L.); 3 Unité de Virologie, Institut Pasteur de Guinée, Conakry BP4416, Guinea; pierre.roques@pasteur.fr; 4 Immunologie des Maladies Virales Auto-Immunes, Hématologiques et Bactériennes (IMVA-HB), Infectious Disease Models and Innovative Therapies (IDMIT): Commissariat a l’Energie Atomique et aux Energies Alternatives (CEA), Institut National de la Santé et de la Recherche Médicale U1184, Université Paris Saclay, 92265 Paris, France; 5 Unité Environnement et Risques Infectieux Groupe Arbovirus, Institut Pasteur, Université de Paris, 75724 Paris, France

**Keywords:** alphavirus, arbovirus, mayaro, favipiravir, antiviral drug

## Abstract

Mayaro virus (MAYV) is an emergent alphavirus that causes MAYV fever. It is often associated with debilitating symptoms, particularly arthralgia and myalgia. MAYV infection is becoming a considerable health issue that, unfortunately, lacks a specific antiviral treatment. Favipiravir, a broad-spectrum antiviral drug, has recently been shown to exert anti-MAYV activity in vitro. In the present study, the potential of Favipiravir to inhibit MAYV replication in an in vivo model was evaluated. Immunocompetent mice were orally administrated 300 mg/kg/dose of Favipiravir at pre-, concurrent-, or post-MAYV infection. The results showed a significant reduction in infectious viral particles and viral RNA transcripts in the tissues and blood of the pre- and concurrently treated infected mice. A significant reduction in the presence of both viral RNA transcript and infectious viral particles in the tissue and blood of pre- and concurrently treated infected mice was observed. By contrast, Favipiravir treatment post-MAYV infection did not result in a reduction in viral replication. Interestingly, Favipiravir strongly decreased the blood levels of the liver disease markers aspartate- and alanine aminotransferase in the pre- and concurrently treated MAYV-infected mice. Taken together, these results suggest that Favipiravir is a potent antiviral drug when administered in a timely manner.

## 1. Introduction

Mayaro virus (MAYV) is a zoonotic arbovirus that is widespread in South America and the Caribbean islands, where it has caused sporadic outbreaks [1,2]. MAYV is the etiologic agent of MAYV Fever and was first isolated in 1954 from five rural workers in southeastern Trinidad (Mayaro county), during an outbreak in which many cases of arthralgia were reported [3]. The isolation of the virus from a five-year-old boy in Haiti in 2015 indicated that MAYV had migrated to the Caribbean and was actively circulating [4,5]. 

MAYV is usually transmitted by the bite of an infected tree-dwelling mosquito, *Haemagogus janthinomys*, which is considered the main vector [2] MAYV is a single- stranded RNA-enveloped virus that belongs to the *Alphavirus* genus [6]. The genome encodes five structural proteins: Capsid (C), Envelope (E1, E2, E3), 6K and four nonstructural proteins (nsP1,2, 3,4) [7]. Alphaviruses can be divided into two groups: encephalitic and arthritogenic viruses [8]. The first is known for its neurotropism and the second manifests a tropism for the joints. MAYV is an arthritogenic alphavirus and symptoms of acute infection include headaches, vomiting, fever, diarrhea and rash, which are sometimes associated with myalgia [9]. Most importantly, debilitating arthralgia has been reported in MAYV-infected patients, similar to that reported in patients infected by other arthritogenic alphaviruses, such as Chikungunya virus (CHIKV), Sindbis virus (SINV), O’nyong nyong virus, and Ross River virus (RRV) [10,11,12,13]. Alphavirus-induced arthralgia usually affects the joints of the hands, feet, ankles, and the extremities, and frequently progresses to polyarthritis [14,15,16]. In addition, rare but severe pathologies, including myocarditis, hemorrhagic and neurological complications, have been reported as a result of MAYV infection [17]. Following a mosquito bite, MAYV disseminates into the bloodstream, the spleen and the liver, and then reaches the joints, muscles and bones, where it can progress to a chronic infection [18]. It has been demonstrated in a mouse model that there is a correlation between the severity and chronicity of MAYV infection and age, and the clinical manifestations of arthritogenic alphavirus in human confirm this observation [19,20]. Pro-inflammatory cytokines have been described as being associated with MAYV disease severity in patients and in mouse models. Some of these include MCP-1, IL-2, IL-9, IL-13, IL-7, VEGF, IL-17, and IP-10 [21,22]. Several mechanisms are thought to be involved in alphavirus pathogenesis, including oxidative stress [23,24,25,26]. MAYV has been shown to regulate ROS production in the liver, which had an impact on the immune response and viral replication in a mouse model [23]. Although few clinical data are available on liver pathology due to MAYV, hepatomegaly, has been observed in patients [27]. In mouse models, MAYV has been shown to intensively replicate in the liver [19,28]. Along with hepatomegaly, this could induce liver function impairment and damage, which, as in liver disease, could biochemically result in elevated alanine aminotransferases (ALAT) and aspartate amino transferase (ASAT) [29].

Despite the clinical impact of MAYV infection, no therapeutic drugs or vaccines have yet been approved for this alphaviral disease. The management of the disease relies on antipyretics, nonsteroidal anti-inflammatory drugs, and analgesics [30,31]. Antiviral strategies have been designed for alphaviral infections targeting the virus itself or host factors [30], some of which use inhibitors targeting the different enzymes involved in the viral life cycle, such as viral RNA dependent-RNA Polymerase, viral capping and viral protease inhibitors [32,33].

Antiviral studies to identify molecules that inhibit MAYV infection are scarce and have more often been conducted in vitro [34,35,36]. In a recent study, the efficacy of Favipiravir on MAYV infection was demonstrated in cell cultures [37]. However, the antiviral effect of this molecule is yet to be studied in vivo. Several studies have shown that Favipiravir inhibits arthritogenic alphaviruses, including Western equine encephalitis virus (WEEV) and CHIKV in cells and in vivo in mice [30,38,39]. In a CHIKV-infected C57BL/6 mouse model, Favipiravir (300 mg/kg/day) was effective when administered during the acute phase of infection and not during the chronic phase after CHIKV infection. CHIKV replication was strongly suppressed in joints and serum during the acute phase [30]. In AG129 CHIKV-infected mice, Favipiravir protected the mice from neurological disorders and death [38].

In this study, we evaluated the effect of Favipiravir, administered at different times during infection, on MAYV-infected C57BL/6 mice. We report here that Favipiravir, depending on the kinetics of its administration, exerts a potent antiviral effect against MAYV infection that is accompanied by a decrease in the serum levels of the MAYV-induced hepatic liver enzymes, ALAT and ASAT.

## 2. Materials and Methods

### 2.1. Virus, Cells and Compounds

The African green monkey epithelial kidney cell line Vero-E6, macaque fibroblasts and hamster kidney BHK-21 cells were grown in Dulbecco’s modified Eagle’s medium (DMEM; Invitrogen, France), supplemented with 5% fetal bovine serum (FBS) (Lonza, Switzerland) and 0.5% penicillin/streptomycin. The macaque tendon fibroblasts were obtained as previously described [40]. Briefly, primary fibroblasts were obtained from the tendons of macaques, which were negative for the presence of CHIKV or Dengue virus infection, as assessed by antibodies and PCR evaluation, after necropsy. The tendons were minced and plated in 3 cm diameter Petri dishes saturated with FCS. The tendon pieces were immerged in DMEM, 20% FCS and the explants were cultured at 37 °C, 9% CO_2_ in a water-saturated atmosphere. After 7 to 10 days, the fibroblasts came out from the explants, then the cells were passaged every 3 days in DMEM, 10% FCS. A UVE/MAYV/1954/TT/TC625 strain isolated in Trinidad and Tobago was obtained from EVAg (www.european-virus-archive.com; June 2020). Ribavirin (Sigma, France) was dissolved in DMSO (Sigma, France) at 20 mg/mL aliquoted per 70 µL and stored at −20 °C. Favipiravir, kindly provided by Toyama Chemical (Japan), was dissolved in phosphate buffered saline (PBS) at 60 mg/mL and stored at −20 °C, prior to use. Both drugs were thawed at room temperature and dilutions were performed in culture medium before usage.

### 2.2. Ethics Statement

The mice were housed in the Institut Pasteur’s Animal facilities, according to the French and European regulations on the care and protection of laboratory animals. The Animal Ethics Committee of the Pasteur Institute (CETEA-Institut Pasteur) and the French Ministry of Higher Education and Research (MESR 00762.02) approved all the animal experiments. The macaque fibroblasts were issued from biobanked tissues obtained from euthanized macaques. These studies were approved by the regional animal care and use committee in accordance with European directive 63/2010/EU: “CREEA Ile de France Sud”, Fontenay aux Roses, decision #A08-012 dated 7 July 2008.

### 2.3. Mouse Experiment

Four-week-old, 12–14 g, female immunocompetent C57BL/6 mice purchased from Charles River Laboratories (Saint Germain Nuelles, France) were used in the experiments. The mice were kept in cages with HEPA filters in the BSL3 animal facilities of the Pasteur Institute. They were raised in a pathogen -free environment and enjoyed free access to unlimited drinking water and food.

After anesthesia with an injectable mixture of ketamine (Merial, Lyon, France) and xylazine (Bayer, Berlin, Germany), the mice were injected subcutaneously in the right hind footpad with 10^6^ pfu of MAYV in a final volume of 20 µL. The mice were monitored for weight, clinical signs and viremia. Blood was collected from the caudal vein. Swelling of the inoculated foot was assessed by measuring the height and width of the perimetatarsal area using a caliper (Mitutoyo, Japan) and was calculated as follows:

$$\frac{Wif\times Bif}{Wcf\times Bcf}\times 100$$

where W: Width, B: Breadth, *if*: Inoculated foot and *cf*: Contralateral foot [41].

### 2.4. In Vitro Activity of Favipiravir

Vero cells (2.5 × 10^5^ cells/well), BHK21 cells (1.2 × 10^5^ cells/well) or primary macaque tendon fibroblasts (passage 11, 2 × 10^5^ cells/well), respectively, were seeded in ninety-six welll plates and exposed to MAYV at a MOI of 0.005 (for Vero cells) and 0.002 (for BHK21 and tendon fibroblasts) for 1 h at 37 °C. The cells were then thoroughly washed twice, and 100 µL of culture medium supplemented with the drugs at a concentration between 300 down to 2.5 µg/mL and 200 down to 5 µg/mL (8 wells per dilution) for Favipiravir and Ribavirin, respectively, was subsequently added. The selected MOI consistently infected and killed all cells in 48 h. Sixteen wells without added virus served as a negative control.

The cell cultures were incubated at 37 °C, 5% CO_2_ and cell viability was monitored until 72 hours post-infection (hpi), using crystal violet staining. Viral inhibition and the 50% inhibitory concentration (IC50%) were computed using Prism software version 8.1 (GraphPad Software, La Jolla, CA, USA).

Before the determination of the IC50%, the MAYV stock titer was determined in the various cell types using crystal violet staining at 72 h post-exposure. The observed titers, obtained by using the Käber method [42], were 6.8 × 10^6^; 6.2 × 10^6^; 3.2 × 10^6^ TCID 50%/mL for the Vero-E6, BHK-21 and primary tendon fibroblasts, respectively.

### 2.5. In Vivo Activity of Favipiravir

**Figure 1:**
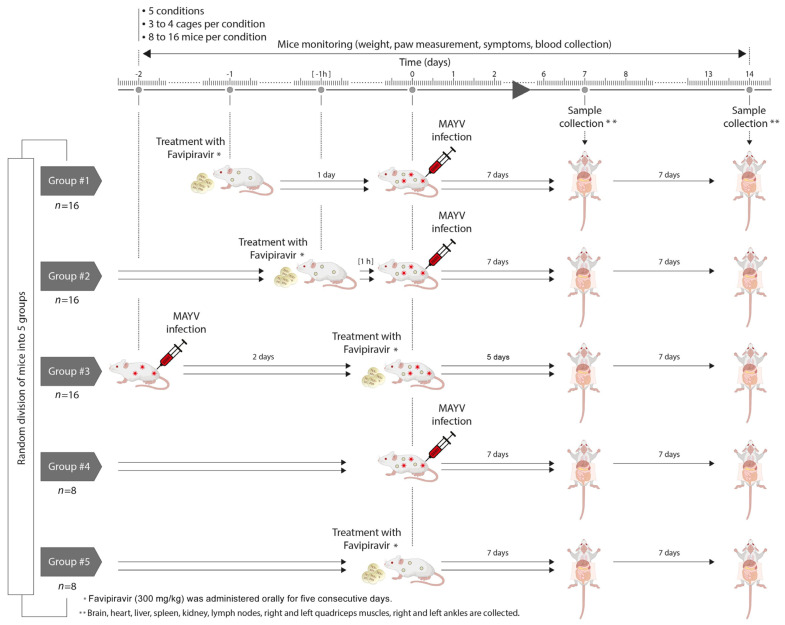
Schematic representation of the experimental procedure.

The mice were randomly divided into five groups, as follows: (1) Pre-treatment: mice (*n* = 16) treated with Favipiravir one day before MAYV infection, (2) Concurrent treatment: mice (*n* = 16) treated with Favipiravir one hour before MAYV infection, (3) Post-treatment: mice (*n* = 16) treated with Favipiravir two days after MAYV infection, (4) CTRL+: MAYV-infected mice (*n* = 8) without treatment and (5) CTRL-: mice (*n* = 8) receiving Favipiravir treatment in the absence of MAYV infection (Figure 1).

The mice were marked on their tails, then housed in cages in groups of three to four in an isolator. Favipiravir (300 mg/kg) was administered orally for five consecutive days. The mice were monitored daily up to 7 days post-treatment and/or exposure to MAYV. Half the number of mice per group were sacrificed after 7 days and the remaining mice 14 days after MAYV infection or Favipiravir treatment.

### 2.6. Tissue Collection

The mice were anesthetized and injected with an intra-cardiac perfusion of PBS. The brain, heart, liver, spleen, kidney, lymph nodes, right and left quadriceps muscles and right and left ankles were collected and kept frozen at −80 °C until use. Serum was separated from the whole blood samples through centrifugation in microvette tubes (Sarstedt, Nümbrecht, Germany), according to the manufacturer’s instructions.

### 2.7. Determination of Viral Spread in Organs and Blood

The mouse organs were dissected at 7 and 14 days post-infection (dpi) to confirm the presence of MAYV RNA and infectious viral particles. The tissues was placed in tissue homogenizing bead tubes (Precellys Lysing kits, Bertin Tech., Montigny-le-Bretonneux, France) with Dulbecco’s modified Eagle’s medium (DMEM). After homogenization, 100 µL of the mixture was transferred to a tube containing RNA extraction lysis buffer. The remaining homogenate was centrifuged and the supernatant was stored at −80 until use. Blood was collected and 20 µL was added to the RNA extraction lysis buffer.

The total RNA was extracted from the organs at 7 and 14 dpi and blood from 0 to 14 dpi using a NucleoSpin RNA Mini kit for RNA purification (Macherey-Nagel, Düren, Germany), according to the manufacturer’s protocol. The extracted RNA was eluted in 50 µL of H_2_O and quantified; 1 µg was used for reverse transcription, using the Moloney murine leukemia virus (M-MLV) reverse transcriptase (Promega, Charbonnières-les-Bains, France). Real-time PCR was carried out using a Maxima probe/ROX qPCR master mix (Promega, France), as described previously [43,44]. The following set of primers and probe were designed and used for MAYV detection: MAYV_F TGCGCCTGCCAGGAGAATGCTGT; MAYV_R TCGCCTGATGCCTTGGCCAACT; FAM-ACGTACCATTGTGTGTGCCCAAT-BHQ.

### 2.8. Detection of Infectious Viral Particles

MAYV titers in the blood and organs were determined by plaque assay, as previously described [44]. Briefly, the Vero-E6 cell line was seeded in triplicate at a concentration of 10^5^ cells per well in a 24 well plate. Serial dilutions of organ homogenates and blood were prepared in DMEM. The inoculum was added to the seeded cells for 1 h and 30 min at 37 °C and 5% CO_2_ while being gently shaken. After incubation_,_ the inoculum was removed and replaced by an overlay medium containing a mix of nutriments and carboxymethyl cellulose (Lonza, Switzerland). The plates were subsequently incubated for four days, after which the cells were fixed with 4% paraformaldehyde and stained with 1% crystal violet. The plates were dried and the plaques were manually determined in each well. The results are presented as plaque-forming units per g (pfu/g) of tissue.

### 2.9. Measurement of ASAT and ALAT Serum Levels

The measurement of aspartate aminotransferase (ASAT) and alanine aminotransferase (ALAT) serum levels were performed by ELISA using aspartate aminotransferase (ASAT) (Elabscience, Houston, USA) and alanine aminotransferase (ALAT) Activity Assay Kits (Elabscience), respectively, according to the manufacturer’s protocol. The results are presented as U/mL. Individual mouse data were used in duplicates.

### 2.10. Statistical Analyses

The statistical analyses were generated with GraphPad Prism 8 software and the experimental results were expressed as mean ± standard deviation (SD). A two-way ANOVA test was used to compare groups and controls. The *p*-values were statistically significant when inferior to 0.05.

## 3. Results

### 3.1. Favipiravir Exerts Antiviral Activity against MAYV In Vitro

**Figure 2:**
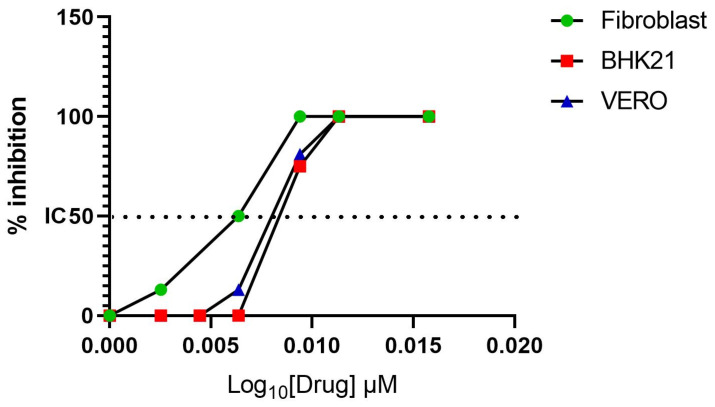
Favipiravir inhibits MAYV in cell culture. Favipiravir was introduced in medium culture 1 h after MAYV exposure of the cells Vero-6, BHK-21 and primate tendon primary fibroblasts at a MOI of 0.005 and 0.002, respectively. Favipiravir MAYV Inhibitory Concentration 50% was obtained on two independent assays and in octoplicates for each dilution of the drug tested.

The Vero-E6 and BHK-21 cells were exposed to MAYV using MOI, which eventually killed all the cells, before being treated with different concentrations of Favipiravir. The inhibition of viral replication was observed in the Vero-E6 and BHK-21 cell types at an IC50% of 0.12 ± 0.01 µM and 0.2 ± 0.001 µM, respectively. Given that tendon fibroblasts are one of the main target cells for MAYV, the same experiment was conducted with the latter cells and the inhibition of viral replication was observed at IC50% of 0.06 ± 0.03 µM (Figure 2). By contrast, Ribavirin did not exert antiviral activity against MAYV replication (data not shown).

### 3.2. MAYV Is Susceptible to Favipiravir In Vivo

**Figure 3:**
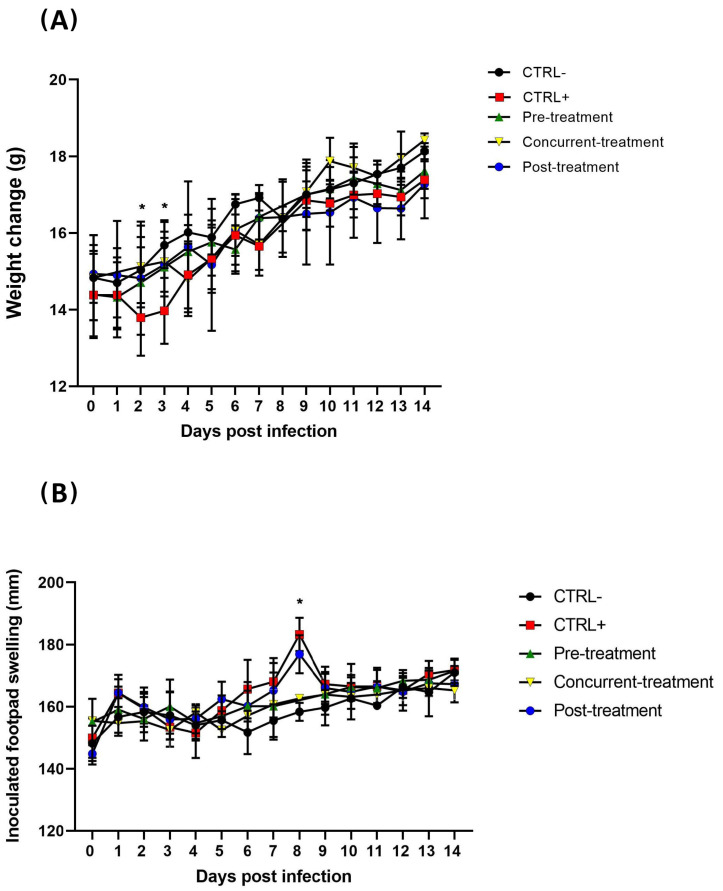
Favipiravir protected against body weight loss and footpad swelling in MAYV-infected mice. MAYV-infected mice (10^6^ pfu) were either treated with Favipiravir at different times of infection or not treated at all. (**A**) Body weight and (**B**) footpad swelling. Numbers of mice were variable within groups as some were sacrificed at 7 dpi and the remaining at 14 dpi. * represents *p* < 0.05, two-way ANOVA was used for statistical analysis. Error bars indicate standard deviation.

Favipiravir was administered to the mice to test whether the in vitro results would translate into an in vivo model by protecting the animal from MAYV infection. For this purpose, a time-course study was carried out to determine the kinetics of MAYV inhibition in C57B/L6 mice during infection (Figure 3). A non-treated group of mice exposed to MAYV (CTRL^+^) and a non-infected group (CTRL^-^) were used as controls. Favipiravir was orally administered at 300 mg/kg/day at pre-, concurrent- and post-MAYV infection for five days and the mice were euthanatized at 7 and 14 dpi. All the mice were injected with 10^6^ PFU of MAYV through the right hind paws, which was not lethal (results not shown). All the mice were weighed and their paws were measured before and every other day after the Favipiravir challenge (Figure 3A,B). A significant weight loss (*p* < 0.05) was noticed at 2 and 3 dpi in the CTRL^+^ group, compared to the mice that were treated concurrently with Favipiravir and the CTRL^-^ mice (Figure 3A). In addition, a statistically significant footpad swelling (*p* < 0.05, two-way ANOVA) was observed in the CTRL+ and post-treated mice, as compared to the pre- and concurrent Favipiravir-treated mice at 8 dpi (Figure 3B)

**Figure 4:**
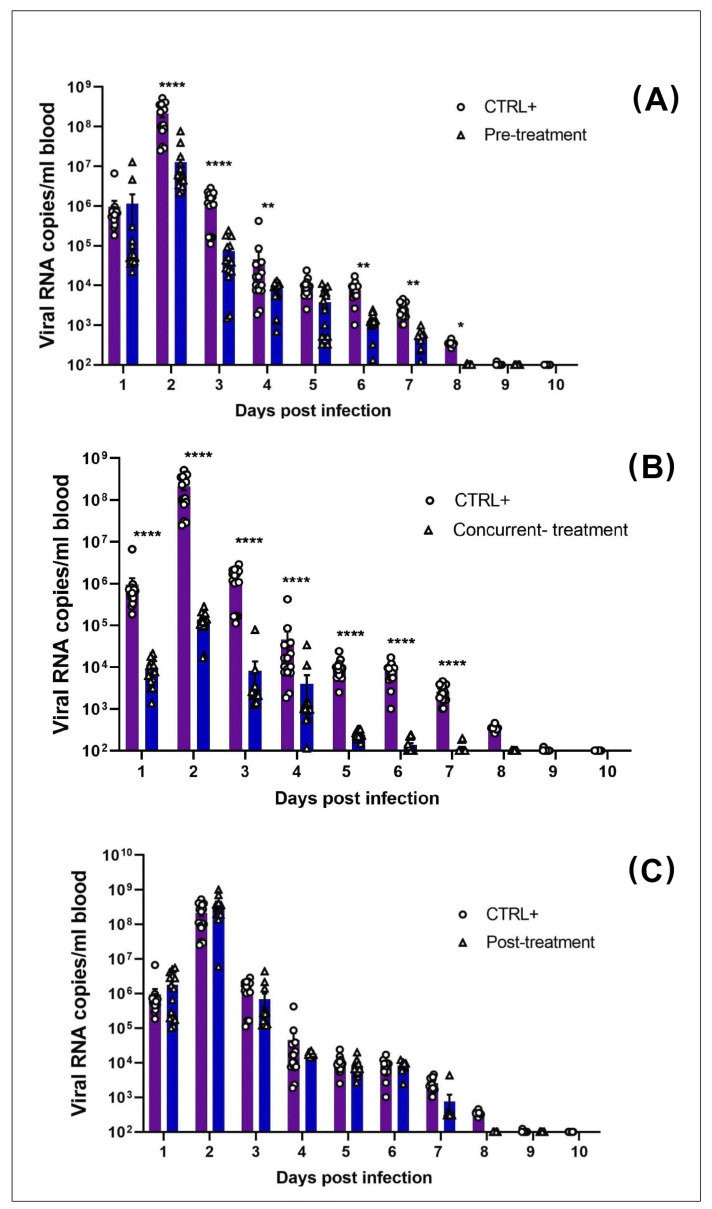

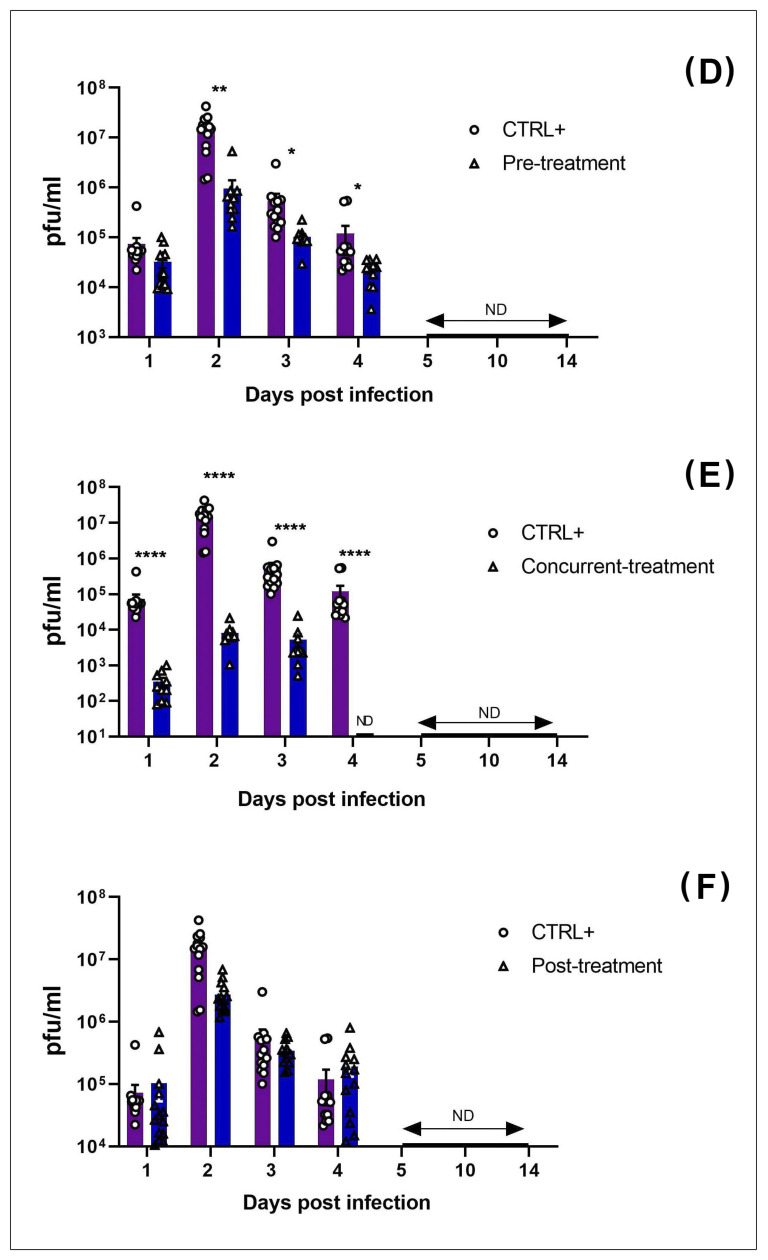
Favipiravir decreased MAYV yield in peripheral blood in pre- and concurrent-treatment. MAYV-infected C57BL/6 mice were orally pre-treated (*n* = 16), concurrently treated (*n* = 16), post-treated (*n* = 16), or untreated (*n* = 8) with Favipiravir 300 mg/kg once daily for 5 consecutive days. Viral RNA and infectious viral particles were quantified in peripheral blood samples using RT-qPCR (**A**–**C**) and plaque assay (**D**–**F**) at different dpi. Each point represents values of an individual mouse. ND (not detected) indicates absence of infectious viral particles in the plaque assay. The two-way ANOVA test and Turkey’s test were used; * *p* < 0.05; ** *p* < 0.01; **** *p* < 0.0001. Half of the mice were sacrificed at 7 dpi and the remaining animals at 14 dpi (see Figure 5).

**Figure 5:**
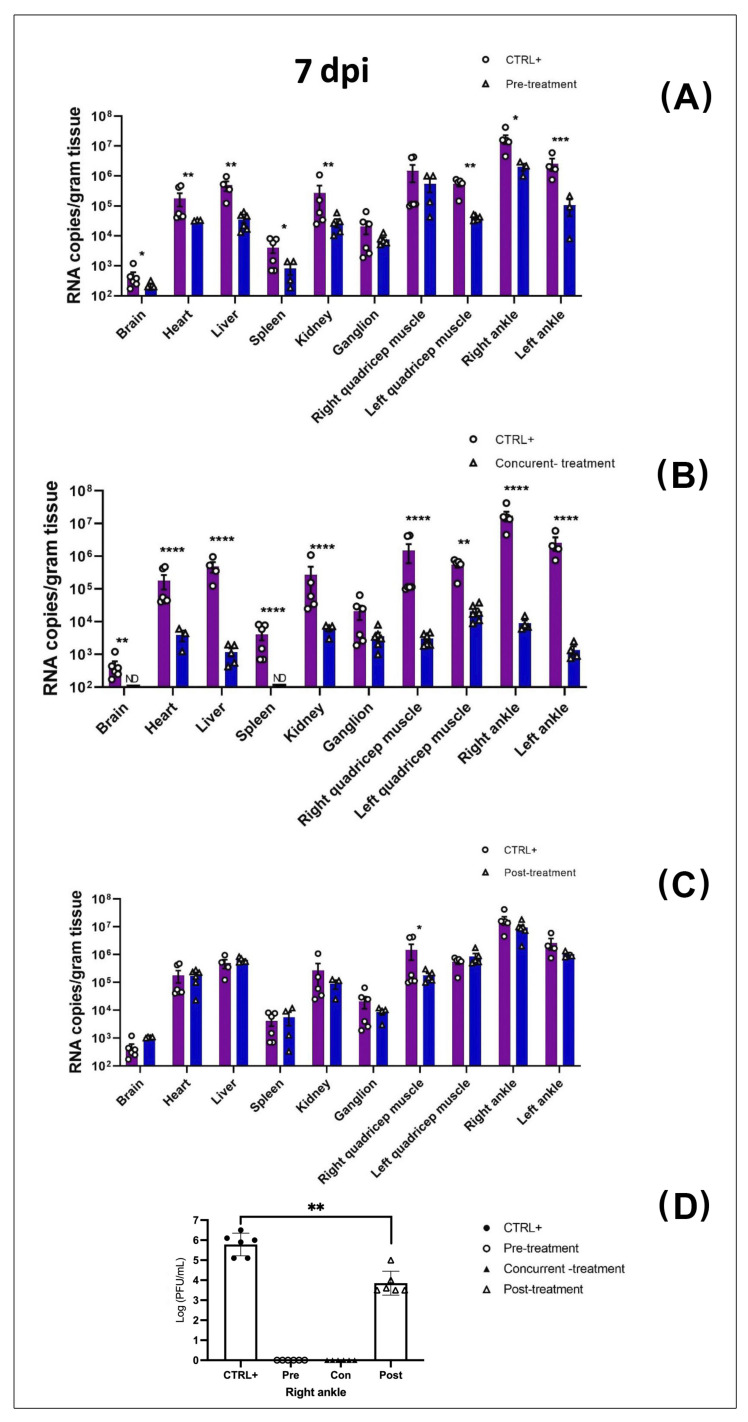

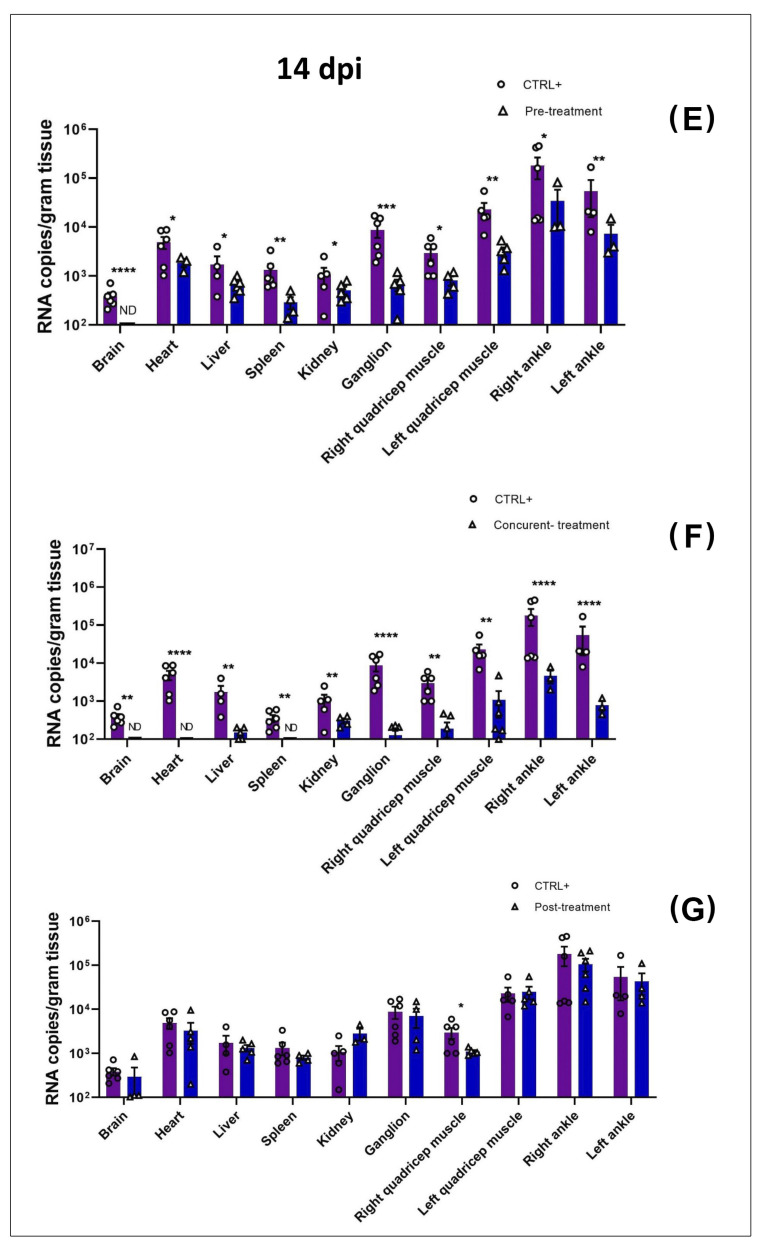
Favipiravir treatment decreased MAYV yield in multiple organs. MAYV-infected four-week-old mice were orally pre-, concurrently and post-treated with Favipiravir 300 mg/kg once daily for 5 consecutive days, or not treated at all. Tissues were harvested and subjected to RT-qPCR and plaque assay for viral load at 7 dpi panel (**A**–**D**) and 14 dpi panel (**E**–**G**). Each treated group (*n* = 4–6) was compared to the MAYV-infected group (*n* = 5). In each tissue graph, dots in the figure correspond to an animal, except in the case of animals with undetectable viral loads (* *p* < 0.05; ** *p* < 0.01; *** *p* < 0.001; **** *p* < 0.0001). ND (not detected) indicates absence of infectious viral particles in the plaque assay. The bar graph is mean ± SD. Data were analyzed with the Mann−Whitney U test to test each group against the other. Each quantification value (dot) in these experiments was the mean of three replicates.

There was a statistically significant decrease in MAYV RNA and infectious viral particles in the blood (Figure 4) and organs (Figure 5) in pre- (Figure 4A,D and Figure 5A,E) and concurrent Favipiravir-treated mice (Figure 4B,E and Figure 5B,F), as compared to the MAYV-exposed mice (CTRL^+^). These differences were more significant between the CTRL^+^ and concurrent treated groups than between the CTRL^+^ and pre-treated groups. In fact, at 2 dpi, there was an approximately three-log difference in the amount of viral copy numbers between the CTRL^+^ and concurrently treated groups that remained significant up to 7 dpi. In addition, with regard to the production of infectious viral particles, this difference was observed until 4 dpi. However, from day 5 onward, in both conditions no infectious viral particles were detected in the blood (Figure 4D–F).

To determine whether pre-, concurrent- and post-treatment with Favipiravir had an antiviral effect on MAYV replication, viral RNA was extracted from several organs/tissues including the brain, quadriceps muscles, and right and left ankles of the infected mice at 7 dpi and 14 dpi.

The results showed a strong decrease in viral RNA in several tissues, especially in the heart, liver, kidneys, right quadriceps muscles and ankles in the concurrently treated mice compared to the CTRL^+^ at 7 dpi (Figure 5B). However, at 7 dpi, there was a more modest reduction in the amount of viral RNA transcripts in all of the organs, except from the left ankle, between the CTRL^+^ and the pre-treatment groups (Figure 5A). Likewise, in the blood, we observed no difference in the number of viral RNA copies between the CTRL^+^ and post-treatment groups; however, there was a statistically significant decrease (*p* < 0.05, two-way ANOVA) in viral RNA in the right quadricep muscles in the post- treated group. Interestingly, infectious viral particles were only detected in the right ankles in the CTRL^+^ and post-treated groups; on the other hand, there were no infectious viral particles detected in the pre- and concurrent Favipiravir-treated groups at 7 dpi (Figure 5D). However, we noticed a significant decrease in infectious viral particles in the post-treated group compared to the CTRL^+^ group (*p* < 0.01) (Figure 5D).

We observed that Favipiravir treatment continued to be effective until day 14 post-MAYV- exposure, particularly in the heart, ganglion and ankles, in the concurrently treated group, compared to the CTRL^+^ group (Figure 5F). For some organs (heart, ganglion, left ankle), the difference between the CTRL^+^ and concurrently treated groups was approximately two logs, while it was roughly one log in the kidney at 14 dpi (*p* < 0.01, Turkey’s test). Apart from the increased number of viral particles in the right quadriceps muscle, there was no significant difference between the CTRL^+^ mice and the post-treatment group (Figure 5G). Finally, no infectious viral particles were detected in any of the groups at 14 dpi (data not shown).

### 3.3. Favipiravir Decreases MAYV-Induced Transaminases

**Figure 6:**
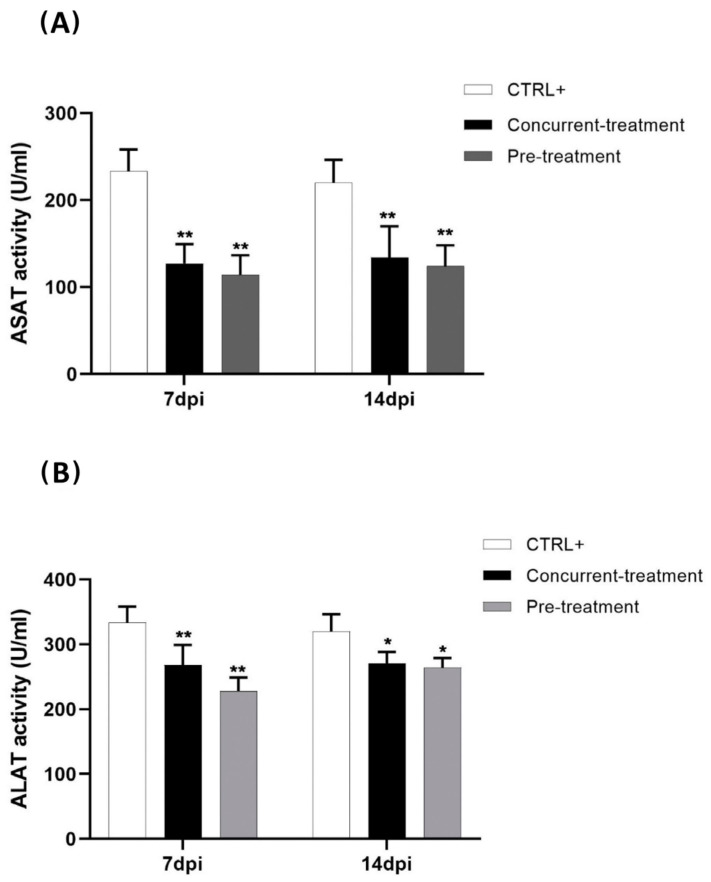
Favipiravir decreases serum ASAT (**A**) and ALAT (**B**) levels in MAYV-infected mice. Hepatic enzyme levels were quantified in MAYV-infected C57BL/6 mice treated (*n* = 4) or not (*n* = 6–8) with Favipiravir at 7 and 14 dpi. The data are expressed as the mean ± SEM. * *p* < 0.05; ** *p* < 0.01.

To evaluate the effect of Favipiravir on MAYV-induced hepatic damage in infected C57BL/6 mice, the serum levels of (ASAT) and (ALAT) in the pre- and concurrently treated mice were quantified at 7 and 14 dpi (Figure 6 A,B). We demonstrated a significant decrease in the amount of ASAT (*p* < 0.01) and ALAT (*p* < 0.01; student’s test) at 7 dpi between the pre-treated and concurrently treated mice compared to the CTRL+ group (MAYV-infected and untreated mice). This difference was also observed at 14 dpi for ASAT (*p* < 0.01) and was less pronounced for ALAT (*p* < 0.05). These results demonstrate that Favipiravir’s effect on viral replication included lowering the hyper-expression of the hepatic enzymes observed in the MAYV-infected mice.

## 4. Discussion

MAYV is known to cause self-limiting illness. However, an incidence of more than 50% of arthralgia/myalgia in infected individuals was reported during several outbreaks in South America [18,21,45]. Knowledge of MAYV’s pathogenesis is based principally on results from relevant animal models and other arthritogenic alphavirus studies and is therefore not fully understood. Following a mosquito bite, MAYV disseminates in the bloodstream and, via the spleen and the liver, it reaches the joints, muscles and bones, where it can cause chronic infection [2,17,46]. At present, no effective treatment or antiviral drugs have been approved or commercialized. The only treatment option currently available is to reduce pain with analgesics and/or non-steroidal anti-inflammatory drugs [17,27]. The co-circulation of other arboviruses in South America and the Caribbean, such as dengue virus and CHIKV, which cause symptoms similar to those of MAYV infection, renders the clinical diagnosis of MAYV fever difficult [5]. In the present study, we hypothesized that because of the reported antiviral activity of Favipiravir against CHIKV [30,38] and several other alphaviruses [39,47], it is Favipiravir is also likely to inhibit MAYV replication in vitro and in vivo. The other objective of this study was to determine the time of administration at which Favipiravir was most effective at decreasing viral infection in vivo.

In the present study, we show that Favipiravir, an RNA polymerase inhibitor [48], strongly decreases MAYV replication both in vitro and in vivo.

MAYV disease is known for its debilitating effects on the joints. Interestingly, in vitro, we found that IC50% seems to be more effective in tendon fibroblast than in reference cell lines. Notably, unlike Favipiravir, Ribavirin demonstrated no anti-MAYV activity in vitro, which corroborates the results in a previously published report conducted by Langendries et al., in which several molecules, including Ribavirin and Favipiravir, were tested for their ability to protect permissive cells against MAYV in vitro [37].

In vivo, our results also demonstrate that the administration of Favipiravir, both prior to and during infection, reduced viral replication and, consequently, decreased footpad swelling at the virus’ inoculation site. It can be inferred that the reduction of infectious viral particles, as well as viral RNA, by Favipiravir correlates with the reduction of arthritis/arthralgia because there was a considerable reduction in footpad swelling in the pre-treated and concurrently treated mice, as compared to the CTRL^+^ mice. However, under the conditions used in this study, this effect was mainly observed when Favipiravir was administered prior to or during infection, whereas a reduction in infectious viral particles was only observed in the right ankles of the post-treated mice. These results suggest that once the infection is established, it can no longer be controlled by Favipiravir at the administered dose and the amount of virus (10^6^ PFU) used for infection. As Favipiravir targets viral RNA polymerase, it can inhibit new viral cycles; the treatment performed at 2 dpi could therefore not be effective compared to its use in the other conditions.

However, we observed intracellular viral RNA at 7 dpi, which decreased at 14 dpi in each of the organs and in all of the infected mouse groups. This was probably due to the animal model used in this study, as immune-competent mice are able to control viremia efficiently at 5 dpi. In addition, the viral titration of MAYV in all the organs at 7 dpi revealed the clearance of the infectious viral particles except, in the infected right ankles of the MAYV-infected and post-treated mice. The persistence of the virus in the ankles of the MAYV-infected and post-treated groups could have been due to the proximity of the site of inoculation, viral evasion and/or host defense mechanisms.

Presumably, cellular infiltrates, such as those involving macrophages, could in part explain this persistence, given that these cells are permissive to MAYV infection, thereby contributing to the continuation of viral replication. Furthermore, even after treatment with Favipiravir, we did not observe a complete clearance of MAYV RNA in the majority of the organs. This observation could be due to the difference between the viral load of MAYV, 10^6^ PFU, with which we inoculated our mice and the load used in the study by Abdelnabi et al. (2018), in which they used 10^3^ PFU of CHIKV [30]. The amount of MAYV used in the current study could have masked the antiviral effects of Favipiravir in the pre- and post-treated groups, thereby preventing the total clearance of MAYV RNA in the organs.

Lethal mutagenesis has recently been considered as an important antiviral strategy against RNA viruses and it is now known that Favipiravir has the ability to elicit this phenomenon by increasing the number of mutations [49,50]. This mutagenic activity, as previously demonstrated with CHIKV and West Nile virus, could also explain how Favipiravir inhibits MAYV replication, as shown in our study.

A wide range of studies has demonstrated the critical role of the liver in the pathogenesis of many viruses, including alphaviruses [51,52,53,54,55]. Camini et al. demonstrated that oxidative stress, along with liver injury, was induced by MAYV infection in the HepG2 cell line, as well as in vivo in BALB/c mice [23,28]. Based on these findings, we hypothesized that MAYV could replicate in the liver, thereby causing organ damage, and that this condition could be prevented or improved by the administration of Favipiravir. Our study revealed a reduction in MAYV infectious viral particles and viral RNA in the livers of pre- and concurrent Favipiravir-treated mice at 7 and 14 dpi, demonstrating that the drug could be beneficial in the case of alphavirus-induced liver disease.

The reduction in viral replication in the liver by Favipiravir was associated with the decrease in serum ALAT and ASAT. Interestingly, the serum levels of these transaminases were found to be considerably decreased in the pre- and concurrently treated mice, as compared to the MAYV-infected mice without treatment, indicating that Favipiravir could protect the animals from potential liver injury due to MAYV liver infection.

The mouse model used in the present study seems to be ideal to study the efficacy of drugs against MAYV infection, like the model previously developed for CHIKV [30,56], because the virus induces clinical signs similar to those observed in MAYV-infected patients and does not cause lethality. By contrast, immunodeficient models, such as IFN receptor-knock-out mice, known to present immature immune systems, are lethal to CHIKV [57], as well as MAYV [19] infection, as a consequence, they are not suitable for these studies. It would be of interest to evaluate the effect of Favipiravir administrated using various doses up to 1400 mg/Kg/day, as described by Driouich et al. 2021 [48], using the present model, when administered during the chronic phase of MAYV infection, which is more representative of the debilitating state of infected patients. Finally, it would be of interest to compare the results obtained from the present mouse model with those obtained from non-human primate models, which are reportedly the most effective for studying alphavirus pathogenesis and drug treatments [40,54].

In conclusion, the limited number of available therapeutic strategies to treat arboviruses is one of the major challenges facing public health. Altogether, our data suggest that Favipiravir, a RNA-Pol inhibitor, could significantly reduce MAYV replication in a majority of the organs targeted by the virus, including the joints, which are the main site of MAYV-induced chronic arthralgia.

## Data Availability

Not applicable.
